# Antiplasmodial Activity Is an Ancient and Conserved Feature of Tick Defensins

**DOI:** 10.3389/fmicb.2016.01682

**Published:** 2016-10-24

**Authors:** Alejandro Cabezas-Cruz, Miray Tonk, Anne Bouchut, Christine Pierrot, Raymond J. Pierce, Michalis Kotsyfakis, Mohammad Rahnamaeian, Andreas Vilcinskas, Jamal Khalife, James J. Valdés

**Affiliations:** ^1^Institute of Parasitology, Université Lille, CNRS, INSERM, CHU Lille, Institut Pasteur de Lille, U1019 – UMR 8204 – Centre d’Infection et d’Immunité de LilleLille, France; ^2^Institute of Parasitology, Biology Centre of the Czech Academy of Sciences (ACVR)České Budějovice, Czech Republic; ^3^Faculty of Science, University of South BohemiaČeské Budějovice, Czech Republic; ^4^Department of Bioresources, Fraunhofer Institute for Molecular Biology and Applied EcologyGiessen, Germany; ^5^Institute for Insect Biotechnology, Justus-Liebig-University of GiessenGiessen, Germany; ^6^Department of Virology, Veterinary Research InstituteBrno, Czech Republic

**Keywords:** ticks, defensins, antimicrobial spectrum, ancestral sequence reconstruction, *Plasmodium falciparum*

## Abstract

Ancestral sequence reconstruction has been widely used to test evolution-based hypotheses. The genome of the European tick vector, *Ixodes ricinus*, encodes for defensin peptides with diverse antimicrobial activities against distantly related pathogens. These pathogens include fungi, Gram-negative, and Gram-positive bacteria, i.e., a wide antimicrobial spectrum. Ticks do not transmit these pathogens, suggesting that these defensins may act against a wide range of microbes encountered by ticks during blood feeding or off-host periods. As demonstrated here, these *I. ricinus* defensins are also effective against the apicomplexan parasite *Plasmodium falciparum*. To study the general evolution of antimicrobial activity in tick defensins, the ancestral amino acid sequence of chelicerate defensins, which existed approximately 444 million years ago, was reconstructed using publicly available scorpion and tick defensin sequences (named Scorpions-Ticks Defensins Ancestor, STiDA). The activity of STiDA was tested against *P. falciparum* and the same Gram-negative and Gram-positive bacteria that were used for the *I. ricinus* defensins. While some extant tick defensins exhibit a wide antimicrobial spectrum, the ancestral defensin showed moderate activity against one of the tested microbes, *P. falciparum*. This study suggests that amino acid variability and defensin family expansion increased the antimicrobial spectrum of ancestral tick defensins.

## Introduction

Pauling and Zuckerkandl (1963) proposed that sequences of molecules from extant organisms can be used to infer sequences that were present in their common ancestors. In this pioneering work, they envisaged that “*one will be able to study the physico-chemical properties of these* (ancestral) *molecules and to make inferences about their functions*.” The hypothesis of ancestral reconstruction has been experimentally tested, for example Hillis et al. (1994) evolved bacteriophage T7 phylogenies in the laboratory and the ancestral T7 sequences were reconstructed with 98–100% accuracy (i.e., the reconstructed T7 sequences were 98–100% of the time identical to the actual ancestors). Subsequently, the statistical methods to infer such ancestral sequences have been improved (Yang et al., 1995; Pupko et al., 2000; Nielsen, 2002), prediction has become more accurate (Thornton, 2004; Harms and Thornton, 2010) and chemically synthesized peptides are routine. This permits a study of ancestral molecules using widely available methods in scientific research.

Using this approach, interesting insights into molecular evolution have been gained, for instance, on lysozymes (Malcolm et al., 1990), ribonucleases (Jermann et al., 1995), thermophilic proteins (Akanuma et al., 2015), and hemoglobin in pikas (Tufts et al., 2015). These ancient molecules not only exhibit the molecular properties of their extant relative, but also are functionally active (Malcolm et al., 1990; Jermann et al., 1995). Ancestral defensin reconstruction was used to study the coordination of amino acid changes in the prodefensin and mature defensin during the evolution of mammalian defensins (Hughes and Yeager, 1997). Chelicerate defensins are a diverse peptide family structurally defined as a Cys stabilized α-helix and β-sheet (CS-αβ) family (Wang and Zhu, 2011; Galay et al., 2012; Tonk et al., 2014b). Selection pressure at the amino acid level has been hypothesized as the diversifying agent for defensins (Hughes, 1999; Gao and Zhu, 2010; Wang and Zhu, 2011). However, the functional implications of such sequence diversification have not been experimentally tested.

We recently identified a diverse family of CS-αβ defensins in the European tick vector, *Ixodes ricinus* (Tonk et al., 2014b). Despite having a conserved CS-αβ tertiary fold, defensins possess substitutions and indels at the primary sequence level within specific functional motifs responsible for bactericidal/fungicidal activity (Zhang and Zhu, 2009). These functional motifs are known as the α-patch, the m-loop and the γ-patch. Tian et al. (2008) identified mutations in the α-patch and the γ-patch between the two *Drosophila melanogaster* defensins drosomycin and drosomycin-2 that dictate their antimicrobial efficacy. We further characterized the activity of these defensins identified from the European tick vector, *I. ricinus*, against different species of fungi, Gram-negative and Gram-positive bacteria that are not transmitted by ticks (Tonk et al., 2015). Testing the activity of tick defensins against microorganisms that are not transmitted by ticks has a twofold utility: (i) it can permit to uncover novel antimicrobial compounds that may be used as an alternative to antibiotic resistant bacteria (Tonk et al., 2014a), and (ii) it will give some clue about how ticks respond to a variety of microorganisms that they may encounter during their life cycle.

Indeed, during off-host periods and blood-feeding, ticks may encounter a variety of microorganisms that may have a detrimental effect on them. We hypothesized that this microbial challenge constitutes a major selection pressure for the diversification and emergence of new functions in the components of the innate immunity of ticks, particularly defensins. Hence, the aim of this study was to test the evolution of antimicrobial activity in chelicerate defensins, specifically ticks. We intended to achieve this by comparing the activity of ancestral chelicerate defensins with that of extant tick defensins. To this aim we reconstructed the sequence of the ancestral chelicerate defensin using available scorpion and tick defensins (named as Scorpions-Ticks Defensins Ancestor, STiDA). We then tested the antimicrobial activity of STiDA against the same bacterial species we tested using the *I. ricinus* defensins. This led us to test the activity of STiDA and *I. ricinus* defensins against the apicomplexan parasite *Plasmodium falciparum*. Using different microbe species belonging to distantly related taxa (bacteria and apicomplexan) allowed us to test and compare the antimicrobial spectrum of ancestral and extant tick defensins. Our results show that STiDA is a defensin peptide with a limited antimicrobial spectrum; it is only active against *P. falciparum*. This implies that expansion of defensin family in ticks and the amino acid diversity of these peptides during evolution may be associated to an expansion in their antimicrobial spectrum. Our comparative structural studies elucidate this antimicrobial spectrum by profiling key functional residues and membrane contacts. The conserved activity of tick defensins against *P. falciparum* highlights an overlooked evolutionarily relation between ticks and the mosquito-borne pathogen *P. falciparum*.

## Materials and Methods

### Phylogenetic Analysis

Scorpion and tick defensins were aligned using MAFFT (Katoh and Standley, 2013). The alignment was used to infer a maximum likehood (ML) phylogenetic tree. The best-fit model of sequence evolution was selected based on the Corrected Akaike Information Criterion (AICc) and the Bayesian Information Criterion (BIC) as implemented in MEGA 6 (Tamura et al., 2013). Accordingly, the LG (Le and Gascuel, 2008) substitution model, which had the lowest values of AICc and BIC, was chosen for subsequent phylogenetic analyses. The ML method implemented in MEGA 6 (Tamura et al., 2013), was used to obtain the best tree topology. All sites of the alignments were used in the tree reconstruction. The proportion of Gamma distributed sites was estimated in MEGA 6 (Tamura et al., 2013). Reliability for internal branches was assessed using the bootstrapping method (1000 bootstrap replicates) implemented in MEGA 6 (Tamura et al., 2013). Graphical representation and editing of the phylogenetic tree was performed with MEGA 6 (Tamura et al., 2013).

### Reconstruction of Scorpions and Ticks Defensin Ancestor

To derive the primary sequence of the chelicerate defensins ancestor, we reconstructed the full amino acid sequence of the ancestor of STiDA using tick (38) and scorpion (26) defensin sequences available in GenBank (Supplementary file 1). The sequences belonged to 14 and 15 different tick and scorpion species, respectively. The reconstruction of the ancestral amino acid sequences was performed using the model-based ML phylogeny above. Three reconstruction methods were used: Marginal (Yang et al., 1995), Joint (Pupko et al., 2000), and Sample (Nielsen, 2002), all of which are implemented in the Datamonkey webserver (Delport et al., 2010). Marginal is a statistical method for reconstructing the amino acid sequence of extinct ancestors, given the phylogeny, and sequences of the extant species. This method assigns character states (amino acids) to interior nodes of the tree using a model of amino acid substitution. Character states having the highest posterior probabilities (calculated by ML) are chosen to reconstruct a particular ancestral site (Yang et al., 1995). Joint reconstruction is an exhaustive algorithm that finds the most likely set of ancestral states in a phylogenetic tree using ML reconstruction (Pupko et al., 2000). Both Joint and Marginal are ML-based methods, however, the ancestral sequence reconstructed using these two methods may differ (Pupko et al., 2000). Finally, Sample uses a variant of ML (the Bayesian approach) to infer ancestral states (Nielsen, 2002). The STiDAs isoelectric points were predicted using ExPASy tools server and their signal peptides were predicted using SignalP 4.1 server (Petersen et al., 2011). Domains were identified using Conserved Domain (Marchler-Bauer et al., 2015) implemented in BLAST.

### Synthetic Peptides

The STiDA sequence information above was used to synthesize the 55 amino acids mature peptides STiDA-1 and STiDA-2 using Fmoc solid phase peptide synthesis (SPPS) with ∼ 95% purity (Pepmic, China). Further details on peptide synthesis are available in Supplementary file 2. Mature peptides were defined as the amino acid sequence obtained after the theoretical cleavage of the furin recognition site (RVRR; Krysan et al., 1999). The synthesis, purification and quality test of *I. ricinus* defensins DefMT2, DefMT3, DefMT5–DefMT7 was done as for STiDA and was previously reported (Tonk et al., 2015).

### Oxidative Folding and Conservation of Peptides

For purification and oxidation of the peptides, the crude product was purified by Reverse Phase High Performance Liquid Chromatography (RP-HPLC; Venusil XBP-C18, 4.6 mm × 250 mm), diluted without drying into a folding buffer (1 M urea, 100 mM Tris, pH 8.0, 1.5 mM oxidized glutathione, 0.75 mM reduced glutathione, 10 mM methionine) and stirred for 48 h at 4°C. Complete oxidation of sulfhydryl groups (-SH) was confirmed using Ellman’s Reagent (Ellman, 1959). The folded, fully oxidized peptides were further purified from the folding mixture by RP-HPLC (Venusil XBP-C18, 4.6 mm × 250 mm) and characterized by Electrospray Ionization Mass Spectrometry (ESI-MS). Accordingly, the folded and oxidized peptides displayed a lower molecular weight (see results). Lyophilized peptides were stored at –20°C until needed.

### Antibacterial Assays

The peptides were added to the culture at different concentrations ranging from 0.015 to 250 μM (final concentrations) and the antimicrobial activity was determined against the Gram-positive bacteria *Listeria monocytogenes* (DSM 20600), *L. fleischmannii* (DSM 24998), *L. grayi* (DSM 20601), *L. marthii* (DSM 23813), *L. innocua* (DSM 20649), *L. welshimeri* (DSM 20650), *L. seeligeri* (DSM 20751), *Staphylococcus aureus* (DSM 2569) and *S. epidermidis* (DSM 3269), and the Gram-negative species *Escherichia coli* (D31) and *Pseudomonas aeruginosa* (DSM 50071). The assays were carried out in 384-well plates (Griener Bio One, Frickenhausen, Germany) using Brain Heart Infusion Broth (BHIB) medium for *Listeria* spp. or Tryptic Soy Broth (TSB; Roth, Karlsruhe, Germany) for the other bacteria. Cultures in the mid-logarithmic phase were used for growth inhibition assays. The initial optical density (OD), measured at a wavelength of 600 nm (OD_600_), for *Listeria* spp. was set to 0.01 and for the rest of the bacteria to 0.001. Changes in OD_600_ values were monitored every 20 min for 24 h using an Eon Microplate Spectrophotometer (BioTek Instruments, Winooski, VT, USA). Each assay also included a medium-only control. Antimicrobial activity was tested against all bacteria and the assays were carried out at least twice with comparable results.

### Growth Inhibition Assay of *Plasmodium falciparum*

The growth inhibition assay was described in detail in Fréville et al. (2013). Briefly, assays were carried out in 96-well plates with a starting parasitemia of 0.5% at a haematocrit of 1% using SYBR Green I (Kelly et al., 2009). The peptides were added to the culture at different concentrations ranging from 6.25 μM to 50 μM (final concentrations) in a volume of 250 μl of RPMI-AlbuMAX (0.5%) and incubated for an additional 48 h to allow all stages to complete one cycle. Cultures were stained for 30 min in the dark with SYBR Green I 1X (Invitrogen) diluted in 20 mM Tris pH8.8, 138 mM NaCl, and fixed with 1% paraformaldehyde. Fixed parasited red blood cells (RBC) were stored at 4°C in the dark until flow cytometry analysis. Parasite growth was assessed by flow cytometry on a BD FACSCantoII (BD Biosciences). Cell pairs were excluded from the analysis using a forward scatter (FSC)-width versus FSC-area dot plot. Infected and uninfected erythrocytes were gated on the basis of their FSC and side scatter (SSC) signals. Fluorescence analysis (Green fluorescence) was performed using BD FACSDiva software (version 6.1.3, BD Biosciences) on a total of 200,000 acquired events. Fluorescence was observed as described by Izumiyama et al. (2009) on a two-parameter dot plot (Green fluoresecence-FSC). Fluorescence of non-infected RBC was adjusted to plot between 100 and 102. Results are expressed as the percentage of growth inhibition.

### Statistical Analysis

The non-parametric Kruskal–Wallis test was used to test the null hypothesis, i.e., that the medians of all groups were equal. Groups were defined as the ‘percent of inhibition’ for *P. falciparum* growth at different peptide concentrations. This test is implemented in the GraphPad 6 Prism program (GraphPad Software Inc.). Differences were considered significant when *P* < 0.05.

### Structural Predictions and Evolutionary Trace Analyses

**FIGURE 1:**
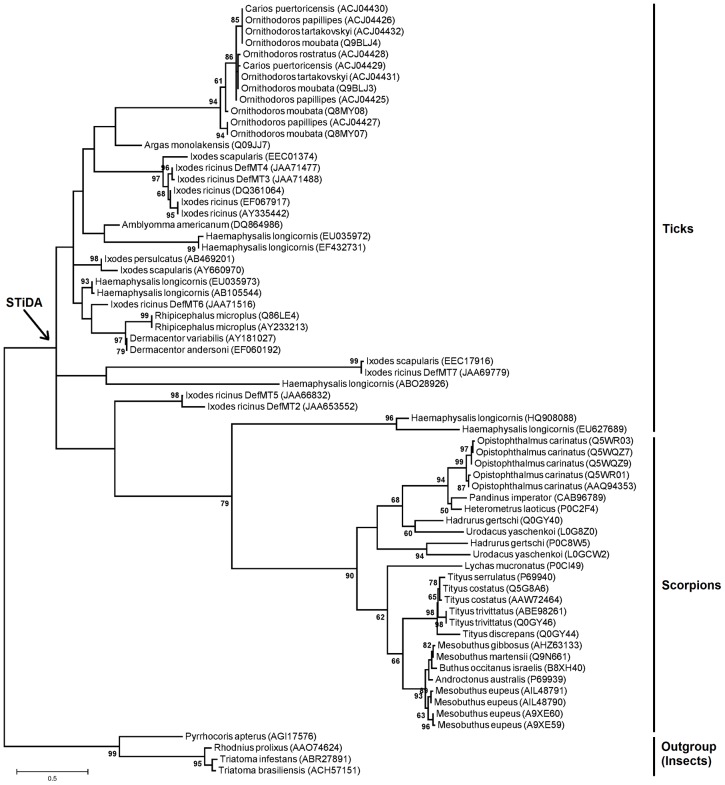
**Phylogenetic tree of defensins and position of Scorpions-Ticks Defensins Ancestor (STiDA).** The maximum likehood (ML) phylogenetic tree was built using amino acid sequences of defensins from ticks and scorpions (Supplementary file 1). Names of species are shown. All sequences were used to predict the sequence of STiDA; (**Figure 2**). The phylogenetic position of STiDA is shown (arrow). Defensins from the insects *Rhodnius prolixus* (Accession number: AAO74624), *Triatoma infestans* (Accession number: ABR27891), *T. brasiliensis* (Accession number: ACH57151), and *Pyrrhocoris apterus* (Accession number: AGI17576) were used as outgroup. Numbers on internal branches are the bootstrap values (1000 replicates).

The server WebLogo (Crooks et al., 2004) was used to graphically represent sequence conservation between STiDA and the tick defensins in this study. All tertiary structures were modeled using the Robetta server^1^ (Raman et al., 2009) and prepared using the Schrodinger’s Maestro software package (Schrödinger, 2010). The structural alignment, secondary structure, residue isoelectric/hydrophobicity, (Multiple Sequence Viewer), and the electrostatic potential (the Possion–Boltzman equation) for each structure were calculated using programs implemented in the Schrodinger’s Maestro software package (Schrödinger, 2010). The PPM server (Lomize et al., 2012) was used to predict the defensin orientation on the membrane and the combined structure was built using Desmond implemented in the Schrodinger’s Maestro software package (Schrödinger, 2010). All tick defensin sequences from **Figure 1** (plus STiDA) were used to identify evolutionary trace key residues for functionally characterized defensins focused in this study. Evolutionary trace was conducted using the server Evolutionary Trace Server (TraceSuite II; Innis et al., 2000) and the predicted tertiary structures of STiDA, DefMT2, and Scapularisin 3 were structural templates to determine buried residues.

## Results

### Reconstruction and General Molecular Properties of Scorpion and Tick Defensin Ancestor

**FIGURE 2:**
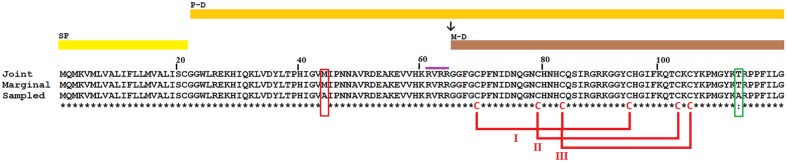
**Amino acid sequence of STiDA obtained by three reconstruction methods.** Three ancestral sequence reconstruction methods were used to predict the amino acid sequence of STiDA, Joint (Pupko et al., 2000), Marginal (Yang et al., 1995), and Sample (Nielsen, 2002). The prediction by Joint and Marginal was identical. However, the prediction by Sample differed in two amino acids (red and green boxes) with that of Joint and Marginal. Conserved pattern of Cys forming putative disulfide bridges is show (numerals). The position of predicted signal peptide (SP), prodefensin (P-D), and mature defensin (M-D) are marked by colored bars. The conserved Furin recognition site (RVRR; violet bar) and the putative cleavage site (↓) are also shown.

Using scorpion and tick defensin peptide sequences we reconstructed the STiDA, for which the phylogenetic position is shown in **Figure 1**. We used three methods of ancestor reconstruction: Joint (Pupko et al., 2000), Marginal (Yang et al., 1995), and Sample (Nielsen, 2002). A site by site report of posterior probabilities for the three methods is presented in Supplementary file 3. The 78 percent of the sites had a posterior probability >95 in the three methods. All three predicted STiDA sequences were 119 amino acid long cationic defensins (theoretical isoelectric point 9.75). The amino acid sequences of STiDA reconstructed by Joint and Marginal were identical (hereafter referred as STiDA-1). However, the sequence of the Sample reconstructed STiDA (hereafter referred as STiDA-2) differed by two amino acids with that of STiDA-1 (**Figure 2**). One substitution was in the N-terminus of the Pro-defensin (position 44, Met → Ala) and the other at the C-terminus (position 113, Thr → Ala). In general, both STiDAs exhibited typical molecular properties of the CS-αβ defensin family, suggesting that these structural properties were established early during the evolution of chelicerates defensins. Using the SignalP 4.1 Server (Petersen et al., 2011), the first 21 amino acids were predicted as a signal peptide, indicating that STiDA was most likely a secreted peptide. The furin cleavage site (RVRR) described by Krysan et al. (1999) was present, as well as the six conserved Cys residues that form the three disulfide bridges (**Figure 2**). In addition, an alignment of STiDA with tick and scorpion defensins (available in Supplementary file 1) shows the six conserved Cys residues (**Supplementary Figure S1**). After theoretical furin cleavage, the mature peptide was 55 amino acids. In agreement with the above conserved molecular properties, the Conserved Domain Database server (Marchler-Bauer et al., 2015) classified STiDA as a member of the Defensin-2 family (pfam01097) with an *E*-value 6^-04^. The BLAST hits with the lowest *E*-values (3^-18^ and 5^-16^, respectively) were produced with defensins from the scorpion *Heterometrus laoticus* (GenBank accession number: P0C2F4) and the tick *Haemaphysalis longicornis* (GenBank accession number: ABW08118). To further explore the relationship of STiDA with extant scorpion and tick defensins, we performed a pairwise comparison of STiDA and the tick and scorpion sequences used for ancestral sequence reconstruction. The top 5 tick and scorpion identities are shown in **Table 1**. The highest identity of STiDA (60.81%) was with a defensin from the tick *H. longicornis*.

**Table 1:** Tick and scorpion defensins with the highest percent of identify with Scorpions-Ticks Defensins Ancestor (STiDA).

Species	Sequence accession number	Identity (%)
*Haemaphysalis longicornis*	AB105544	60.81
*Ixodes persulcatus*	AB469201	56.76
*Amblyomma americanum*	DQ864986	56.94
*Dermacentor andersoni*	EF060192	55.41
*Tityus costatus*	Q5G8A6	56.32


### Production and Folding of Synthetic STiDA

In general, chelicerate synthetic defensins are active as mature peptides (Nakajima et al., 2003; Isogai et al., 2009, 2011; Wang et al., 2015). Therefore, mature STiDA1 and 2 (55 amino acids each), synthesized by Fmoc SPPS with ∼95% purity, were refolded in folding buffer to obtain the peptides in their thermodynamically most stable form. Final purification of folded peptide was achieved by Reverse Phase High Performance Liquid Chromatography. ESI-MS and Ellman’s reagent further characterized the folding status. The molecular weight of peptides such as STiDA1 and 2 having three -S-S- bonds should be 6 (molecular weight of hydrogen H^+^ is approximately 1) less than the one before the oxidation, resulting from the loss of 6 H^+^ from the six reduced sulfhydryl groups (–SH). This was confirmed using ESI-MS. While the theoretical molecular weights of linear STiDA1 (Formula: C_268_H_406_N_82_O_71_S_7_) and 2 (Formula: C_267_H_404_N_82_O_70_S_7_) were 6137.08 and 6107.06, the experimental molecular weights determined by ESI-MS after the folding protocol were 6131.13 and 6101.10, respectively (**Supplementary Figure S2**). Quantitative analysis of -SH revealed that no cysteine residues were available for complexing with the Ellman’s reagent, indicating, in combination with the ESI-MS results, that all cysteines were involved in intramolecular disulfide linkages.

### Comparative Antimicrobial Activity of STiDA

**FIGURE 3:**
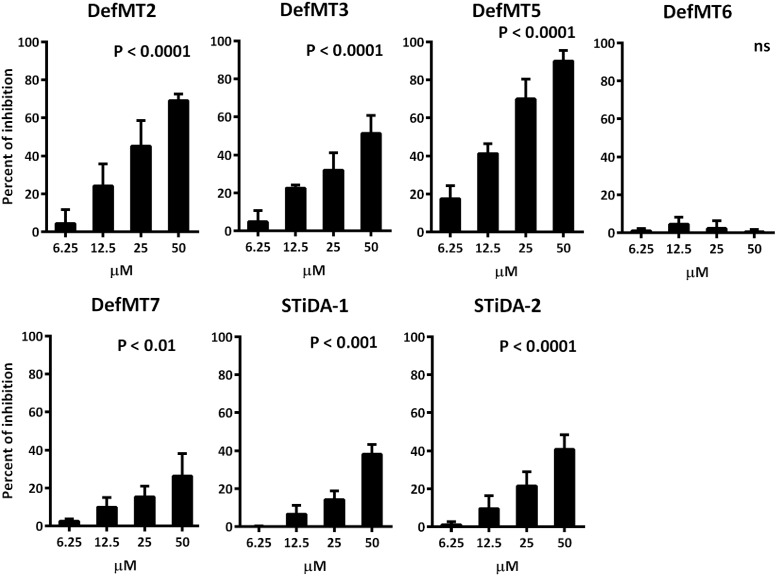
**Anti- *Plasmodium falciparum* activity of STiDA and *Ixodes ricinus* defensins.** The percent of *P. falciparum* growth inhibition induced by STiDA and *I. ricinus* defensins was calculated. Increasing concentrations (6.25–50 μM) of STiDA (1 and 2), DefMT2, DefMT3, DefMT5, and DefMT7 correlate with higher percent of inhibition. No effect was observed using DefMT6. The non-parametric Kruskal–Wallis test was used to test the null hypothesis, i.e., that the medians of all groups were equal. Differences were considered significant when *P* < 0.05.

The antimicrobial activity of STiDA was tested against Gram-positive and Gram-negative bacteria and the apicomplexan parasite *P. falciparum*. STiDA did not show bactericidal activity against any of the tested bacteria. The results of the antibacterial assay for one representative of each bacteria genus are presented in **Supplementary Figure S3**. However, increasing concentrations of both STiDA-1 and STiDA-2 exhibited significant (*P* < 0.001) inhibitory activity on blood stages of *P. falciparum* (**Figure 3**). In order to compare the antimicrobial activity of STiDA with that of extant tick defensins, we determined the activity of recently described *I. ricinus* defensins (Tonk et al., 2014b) against *P. falciparum*. Except for DefMT6, all *I. ricinus* defensins showed significant (*P* < 0.01) inhibitory activity on blood stages of *P. falciparum* (**Figure 3**). From these results, DefMT5 appears to be the most effective against *P. falciparum*. At low concentration (6.25 μM), DefMT5 inhibited the growth of *P. falciparum* by 20%, and at the highest concentration tested (50 μM), it inhibited the growth of this parasite close to 100%. The bactericidal and fungicidal activities of *I. ricinus* and *I. scapularis* defensins were previously reported (Tonk et al., 2014a, 2015). **Table 2** shows that the activity of STiDA (hereafter referring to both STiDA-1 and STiDA-2 as they did not show difference in activity) is comparable to that of *I. ricinus* defensins DefMT2 and DefMT7, which do not have bactericidal activity, but do have antiplasmodial activity.

**Table 2:** Comparative antimicrobial activity of ancestral and extant tick defensins.

	Defensins					
	
Microorganisms	STiDA	DefMT2^∗^	DefMT3^∗^	DefMT5^∗^	DefMT6^∗^	DefMT7^∗^	Scapularisin-3^∗∗^	Scapularisin-6^∗∗^
**Bacteria Gr+**						
*Listeria monocytogenes*	-	-	+	+	+	-	-	-
*L. fleischmannii*	-	-	+	+	+	-	-	-
*L. grayi*	-	-	+	+	+	-	-	+
*L. marthii*	-	-	-	-	-	-	-	-
*L. innocua*	-	-	-	-	-	-	-	-
*L. welshimeri*	-	-	-	-	-	-	-	-
*L. seeligeri*	-	-	+	+	+	-	-	-
*Staphylococcus Aureus*	-	-	+	+	+	-	-	-
*S. epidermidis*	-	-	-	-	+	-	-	-
**Bacteria Gr-**						
*Escherichia coli*	-	-	-	-	+	-	-	-
*Pseudomonas Aeruginosa*	-	-	+	+	+	-	-	-
**Fungi**						
*Fusarium culmorum*	NT	-	+	+	+	-	+	+
*F. graminearum*	NT	-	+	+	+	-	+	+
**Apicomplexan**						
*Plasmodium falciparum*	+	+	+	+	-	+	NT	NT


*^∗^Data collected from (Tonk et al., 2015). ^∗∗^Data collected from (Tonk et al., 2014a). ‘NT’ not tested. ‘+’ activity. ‘-’ no activity.*

### Structural and Functional Properties of STiDA Compared to Extant Tick Defensins

**FIGURE 4:**
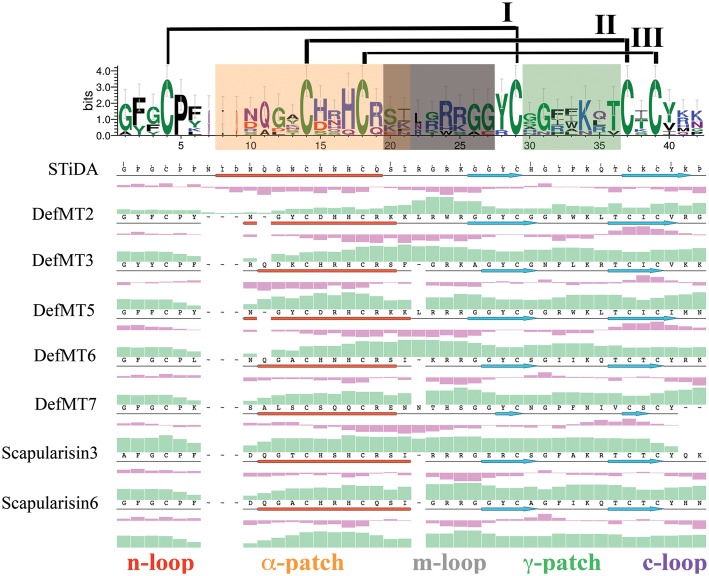
**Sequence-based physicochemical properties of STiDA and extant tick defensins.** The logo above the multiple sequence alignment demonstrates indels and mutations between STiDA and extant tick defensins. The overall height of the stack indicates the sequence conservation, the height of each residue is it relative frequency, and the width of each residue is proportional to its validity, e.g., thin residues have many gaps. The colors indicate the chemical properties of each residue: green = polar; purple = neutral; blue = basic; red = acidic; black = hydrophobic. The error bars are based on a Bayesian 95% confidence interval. The disulfide bridges are noted and ordered in roman numerals. The motifs described by Tian et al. (2008) responsible for antimicrobial/fungicidal activity are equally color-coded throughout the figure and labeled as α-patch (within the α-helix; orange), the m-loop (the α-helix/β-sheet loop; gray), and the γ-patch (theβ-hairpin loop; green). The position of the α-helices (depicted as red cylinders) and β-sheets (depicted as blue arrows) are shown below the amino acid sequence of each defensin. The n-loop (red) and c-loop (purple) denote the N- and C-terminus, respectively. The secondary structure, hydrophobicity (purple histograms) and isoelectric points (green histograms) are plotted below each sequence of the alignment.

**FIGURE 5:**
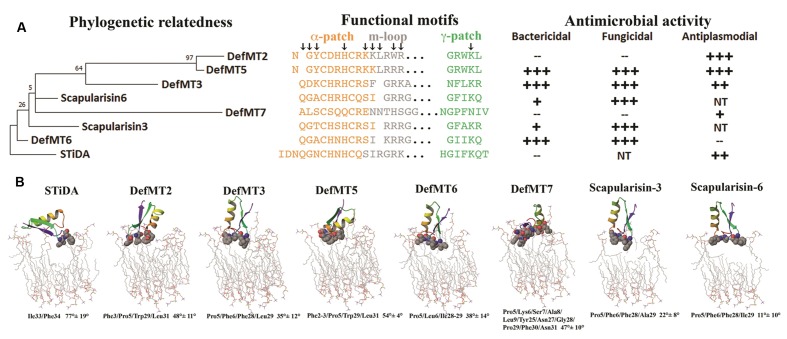
**Structure and function in an evolutionary context of tick defensins and their electrostatic potential.** **(A)** Depicts a rooted ML phylogenetic tree of STiDA and extant tick defensins. The primary sequences of the defensin functional motifs are indicated and the arrows denote the evolutionary traced residues. The antimicrobial properties from **Table 2** are outlined according to their effective concentration +++ = very high; ++ = high; + = low; – = No activity; NT = Not tested. **(B)** Depicts STiDA and each extant tick defensin from this study bound to a membrane. The membranes are shown as sticks and the defensin residues that form contact are in spheres. The atoms are color-coded as carbon = gray, oxygen = red, nitrogen = blue, and phosphorous = purple. Hydrogen atoms are not shown. The specific contacts residues (and respective sequence position) and the defensin orientation (given in degree ±SE) are indicated below each structure.

Tick defensins belong to the CS-αβ family (Wang and Zhu, 2011; Galay et al., 2012), but possess substitutions and indels at the primary sequence level within the functional motifs. The functional motifs α-patch (within the α-helix), the m-loop (the α-helix/β-sheet loop) and the γ-patch (theβ-hairpin loop) are equally color-coded in **Figures 4** and **5**. Except for the conserved Pro5 and the six Cys residues (that form the three disulfide bridges), the sequence logo in **Figure 4** shows few residues within the functional motifs with a higher frequency (≥ 2 bits) between STiDA and the extant tick defensins. These include residues His15/17, Arg19/24, Tyr28, Lys34, and Thr36 (numbered according to the STiDA positions). A clear distinction between STiDA and extant tick defensins is the deletion of the first few residues of the α-helix (**Figure 4**). DefMT7 also possesses shorter β-sheets than STiDA and the other extant tick defensins. The functional motifs of STiDA are mainly hydrophilic, except for the hydrophobic Gly31 in the γ-patch. However, the γ-patch of DefMT2 and DefMT5 are increased in hydrophilicity, while DefMT7 has two hydrophobic residues in the α-patch (**Figure 4**). The isoelectric points for each residue within the α-patch and the γ-patch increased in extant defensins compared to STiDA, but decreased within the m-loop of tick defensins DefMT3, DefMT6, and the Scapularisins. These tick defensins also have a deletion at position 22 within the m-loop (**Figure 4**). **Supplementary Figure S4** depicts that the amino acid substitutions and indels also impact on the electrostatic potential surrounding the functional motifs. Except for DefMT7, most extant tick defensins show an increased net positive charge compared to STiDA.

The STiDA rooted ML phylogeny in **Figure 5A** shows the relatedness, specifically between *I. ricinus* defensins DefMT2, DefMT3, and DefMT5. The DefMT6 falls closest to STiDA. Evolutionary tracing, a computational approach used to trace putative functional regions of a protein family (Innis et al., 2000; Tian et al., 2008), indicated key residues within the functional motifs (denoted by arrows in **Figure 5A**) that may be responsible for the differences in antimicrobial activity. The α-patch has five to six key residues that have indels or substitutions compared to STiDA. These include the Gln residue deleted in DefMT2 and DefMT5 and substituted in DefMT7 (Ala), and the substituted Gly in DefMT3 (Asp) and DefMT7 (Leu). The α-patch Asn of STiDA is highly variable among the extant tick defensins, but the His residue is conserved among the extant tick defensins except for DefMT7 (Gln). Of the three functional motifs, the m-loop has the most indels and substitutions (**Figure 5A**). Specifically, are the Trp substitution in DefMT2 and the His in DefMT7 compared to the conserved Arg in the other tick defensin and STiDA. The γ-patch has the key Lys residue that is conserved among the extant tick defensins except for the Asn substitution in DefMT7. Although these evolutionary traced key residues (**Figure 5A**) may determine the specificity of antimicrobial activity, hypothetically, the initial membrane contact(s) formed should elucidate additional comparative distinctions between STiDA and the extant tick defensins.

Despite the fact that some fungal defensins do not form pores on targeted cells (Mygind et al., 2005; Schneider et al., 2010), most defensins (Brogden, 2005; van Dijk et al., 2007; Poon et al., 2014), including tick defensins (Nakajima et al., 2003), target microbial membranes thereby disrupting their integrity. **Figure 5B** shows the initial residue-membrane contacts formed by each defensin in this study. The STiDA only forms membrane contact with two residues from the γ-patch. This γ-patch-membrane contact is common to the extant tick defensins, but the latter also include the n-loop in their contacts. The exception is DefMT7, since it includes additional residue-membrane contacts in the α-patch and c-loop. The n- and c-loops are the peptide termini shown in **Figure 4**. The γ-patch residue pairs of STiDA that form the membrane contacts are Ile and Phe. Some mutations (Leu, Asn, or Ala) have occurred in the γ-patch of extant tick defensins that form membrane contacts, but most possess either an Ile or Phe contact. The exceptions are DefMT2 and DefMT5 (Trp). These contacts have greatly influenced the orientation of the extant tick defensins compared to STiDA. The STiDA aligns perpendicular to the membrane, while the other defensins are more parallel to the membrane. Peptide-membrane orientations are influenced by the presence and location of Phe (Victor and Cafiso, 2001), which may also explain the antimicrobial shifts in **Figure 5A**, for example, the lack of Phe in DefMT6 compared to STiDA (**Figure 5B**). Another example is that both DefMT3 and DefMT5 have two Phe residues that make contact with the membrane, while DefMT2 only has one (**Figure 5B**).

## Discussion

In this study, we reconstructed the ancestral amino acid sequence of scorpion and tick defensins and tested its antimicrobial activity against Gram-positive and Gram-negative bacteria and the apicomplexan parasite *P. falciparum*. STiDA is a putative defensin peptide prototype present in the most recent common ancestor of ticks and scorpions that possibly existed in the Paleozoic era approximately 444 million years ago in the intersection of the Ordovician and Silurian (Jeyaprakash and Hoy, 2009). Our sequence and structure analyses showed that STiDA is a typical defensin. Seven features of extant typical CS-αβ defensins were found to be conserved in this ancestral peptide (Wang and Zhu, 2011; Galay et al., 2012; Tonk et al., 2014b), namely (i) a signal peptide; (ii) the presence of prodefensin sequence; (iii) a Furin cleavage recognition site; (iv) the six Cys motif; (v) the functional motifs α-patch, m-loop and γ-patch; (vi) two C-terminus anti-parallelβ-sheets; (vii) and, an N-terminus α-helix. At the amino acid level, only the six Cys and the Pro after the first Cys were conserved in STiDA and extant tick defensins (**Figure 4**). Considering this high amino acid variability, it is remarkably that the structure conservation of CS-αβ defensins has been maintained over such large evolutionary time. However, Ingles-Prieto et al. (2013) showed that despite considerable sequence differences compared with extant enzymes, Precambrian thioredoxins have small structural changes over four billion years. These results suggest that different protein folds are conserved over long evolutionary times (Yount and Yeaman, 2004; Yeaman and Yount, 2007). Interestingly, the best scorpion hit using BLAST was a defensin from the venom of *H. laoticus* that is both an antimicrobial peptide and a toxin (Uawonggul et al., 2007). This agrees with the hypothesis that ancestral ticks may have been venomous animals (Cabezas-Cruz and Valdés, 2014). A correct folding is important for the biological function of defensins. More sophisticated methods can be used to evaluate the correct folding of defensins (e.g., Circular Dichroism, Veldhuizen et al., 2008). However, the methods described here are standard procedures to test the quality and folding of synthetic defensins for antimicrobial assays (Nakajima et al., 2003; van Dijk et al., 2007; Isogai et al., 2009; Tonk et al., 2015).

The growth inhibition properties of STiDA against *P. falciparum* confirmed that it is an antimicrobial peptide, though with moderate activity and a reduced antimicrobial spectrum since it was not effective against any of the bacteria tested. This suggests that the antimicrobial spectrum of tick defensins expanded during evolution to include the bacteria tested in this analysis, but also that the ancestral peptide may have targeted extant or extinct pathogens that were not tested in this paper or in prior studies. No difference in anti-plasmodium activity was observed in the two predicted STiDA (1 and 2) (**Figure 3**) therefore we concluded that the difference in ancestral sequence reconstruction using Joint and Marginal vs. Sample had minor functional impact, if any. Interestingly, DefMT2 and DefMT7 that were not effective against any of the tested bacteria or fungi species, were effective against *P. falciparum* indicating that specific antimicrobial activity against this pathogen exists in ticks and is evolutionary conserved. This reflects on the effect other tick defensins possess against tick-borne apicomplexan parasites, such as *Babesia* sp. (Tsuji et al., 2007). Another possible explanation is that during blood feeding ticks are exposed to *Plasmodium* spp. and develop a specific response against these pathogens. In agreement with this hypothesis, a previous study showed that during feeding on naturally infected populations of lizards, *I. pacificus* ticks can acquire *P. mexicanum*. However, this tick species did not allow *Plasmodium* replication, while mites did (Schall and Smith, 2006). A time-dependent elimination of *P. mexicanum* was also observed in *I. pacificus* (Schall and Smith, 2006).

Defensins have been reported as sweet-tasting peptides, antimicrobial peptides, and toxins, demonstrating the versatility of the defensin structural scaffolding (Zhu et al., 2005; Mulder et al., 2013; Goyal and Mattoo, 2014). In addition, minimal experimental manipulation at the sequence level transformed a non-toxic defensin into a neurotoxin (Zhu et al., 2014). This reinforces the hypothesis that a small number of amino acid mutations may have major impact on the functional diversification of defensins. Our results provide examples of minor sequence variation that have a major impact on antimicrobial activity in STiDA and the extant tick defensins. DefMT2 was shown to be highly effective against *P. falciparum*, however, it does not show any effect on bacteria (Gram-positive and Gram-negative) or fungi, while DefMT5, which is closely related to DefMT2, is very effective against bacteria, fungi and *P. falciparum* (present results and Tonk et al., 2015). The mature peptides of DefMT2 and DefMT5 have six amino acid substitutions, of which only two are within the functional motifs - the α-patch (His→Arg) and m-loop (Trp→Arg). Amino acid substitutions in these motifs have been suggested to influence the efficacy of defensin fungicidal and antiparasitic activity (Tian et al., 2008). In the m-loop and γ-patch of DefMT6, which is the closest relative of STiDA (**Figure 5A**), one deletion (Arg→0) and three amino acid substitutions (Gly→Lys, Lys→Arg, and Phe→Ile) may explain the shift from the antiplasmodial activity in STiDA to the bactericidal and fungicidal activity in DefMT6. However, from the present study the activity of STiDA against fungi cannot be ruled out because these microorganisms were not included in our activity assays. The deletion (Arg→0) in the m-loop is conserved in defensins from different tick species, *I. scapularis* (Scapularisin-3 and Scapularisin-6) and *I. ricinus* (DefMT3 and DefMT6), suggesting that this deletion occurred before the separation of these two species.

## Conclusion

Hypothetically, natural selection has diversified defensins at the amino acid level. Here we provided experimental evidence that amino acid diversification and family expansion in tick defensins increased their antimicrobial spectrum. An unexpected conserved activity of *I. ricinus* defensins against *P. falciparum* was found indicating that antiplasmodial activity is an ancient and conserved feature of tick defensins. Inferences from both experimental studies and structural analysis, of all three functional motifs, point to key residues that dictate tick antimicrobial efficacy. These include the conserved His residue in the α-patch, the two adjacent positively charged residues in the m-loop, and the key Lys residue in the γ-patch.

## Author Contributions

Conceived and designed the experiments: AC-C, JV, MT, JK, CP, and AB. Performed the experiments: MT and AB. Performed bioinformatic and evolutionary analysis: AC-C and JV. Analyzed the data: AC-C, JV, AB, CP, JK, and MT. Contributed reagents/materials/analysis tools: MR, AV, JK, JV, and RP. Drafted the manuscript: AC-C and JV. All authors read, made a critical revision and approved the final manuscript.

## Conflict of Interest Statement

The authors declare that the research was conducted in the absence of any commercial or financial relationships that could be construed as a potential conflict of interest.

## Footnotes

1 http://robetta.bakerlab.org

## Supplementary Material

**FIGURE S1 | Alignment of Scorpions-Ticks Defensins Ancestor (STiDA) with tick and scorpion defensins used in ancestral sequence reconstruction.**

**FIGURE S2 | Experimental determination of molecular weight for folded and oxidized STiDA1 and 2 using Electrospray Ionization Mass Spectrometry (ESI-MS).**

**FIGURE S3 | Results of antibacterial assay for one representative of each bacteria genus (*Listeria grayi. Staphylococcus epidermidis. Escherichia coli*, and *Pseudomonas aeruginosa*) that were tested.** Increasing concentrations of STiDA (1 and 2) did not inhibit the growth of the tested bacteria after 24 h of incubation in presence of STiDA. Similar results were obtained with the rest of the bacteria species.

**FIGURE S4 | The STiDA (boxed) predicted tertiary structure (N-terminus/Red→ C-terminus/Purple) and its electrostatic potential below (180° turn; blue = positive, white = neutral, and red = negative).** The electrostatic potential for the other extant tick defensins are also represented.

## References

[B1] Akanuma S. Yokobori S. I. Nakajima Y. Bessho M. Yamagishi A. (2015). Robustness of predictions of extremely thermally stable proteins in ancient organisms. *Evolution* 69 2954–2962. 10.1111/evo.12779 26404857

[B2] Brogden K. A. (2005). Antimicrobial peptides: pore formers or metabolic inhibitors in bacteria? *Nat. Rev. Microbiol.* 3 238–250. 10.1038/nrmicro1098 15703760

[B3] Cabezas-Cruz A. Valdés J. J. (2014). Are ticks venomous animals? *Front. Zool.* 11:47 10.1186/1742-9994-11-47 PMC4085379 25006341

[B4] Crooks G. E. Hon G. Chandonia J. M. Brenner S. E. (2004). WebLogo: a sequence logo generator. *Genome Res.* 14 1188–1190. 10.1101/gr.849004 15173120 PMC419797

[B5] Delport W. Poon A. Frost S. Kosakovsky P. (2010). Datamonkey 2010: a suite of phylogenetic analysis tools for evolutionary biology. *Bioinformatics* 26 2455–2457. 10.1093/bioinformatics/btq429 20671151 PMC2944195

[B6] Ellman G. L. (1959). Tissue sulfhydryl groups. *Arch. Biochem. Biophys.* 82 70–77. 10.1016/0003-9861(59)90090-6 13650640

[B7] Fréville A. Cailliau-Maggio K. Pierrot C. Tellier G. Kalamou H. Lafitte S. (2013). Plasmodium falciparum encodes a conserved active inhibitor-2 for protein phosphatase type 1: perspectives for novel anti-plasmodial therapy. *BMC. Biol.* 11:80 10.1186/1741-7007-11-80 PMC3735429 23837822

[B8] Galay R. L. Maeda H. Aung K. M. Umemiya-Shirafuji R. Xuan X. Igarashi I. (2012). Anti-babesial activity of a potent peptide fragment derived from longicin of *Haemaphysalis longicornis*. *Trop. Anim. Health. Prod.* 44 343–348. 10.1007/s11250-011-0027-7 22102016

[B9] Gao B. Zhu S. (2010). Identification and characterization of the parasitic wasp *Nasonia* defensins: positive selection targeting the functional region? *Dev. Comp. Immunol.* 34 659–668. 10.1016/j.dci.2010.01.012 20097222

[B10] Goyal R. K. Mattoo A. K. (2014). Multitasking antimicrobial peptides in plant development and host defense against biotic/abiotic stress. *Plant. Sci.* 228 135–149. 10.1016/j.plantsci.2014.05.012 25438794

[B11] Harms M. J. Thornton J. W. (2010). Analyzing protein structure and function using ancestral gene reconstruction. *Curr. Opin. Struct. Biol.* 20 360–366. 10.1016/j.sbi.2010.03.005 20413295 PMC2916957

[B12] Hillis D. M. Huelsenbeck J. P. Cunningham C. W. (1994). Application and accuracy of molecular phylogenies. *Science* 264 671–677. 10.1126/science.8171318 8171318

[B13] Hughes A. L. (1999). Evolutionary diversification of the mammalian defensins. *Cell. Mol. Life Sci.* 56 94–103. 10.1007/s000180050010 11213266 PMC11147084

[B14] Hughes A. L. Yeager M. (1997). Coordinated amino acid changes in the evolution of mammalian defensins. *J. Mol. Evol.* 44 675–682. 10.1007/PL00006191 9169560

[B15] Ingles-Prieto A. Ibarra-Molero B. Delgado-Delgado A. Perez-Jimenez R. Fernandez J. M. Gaucher E. A. (2013). Conservation of protein structure over four billion years. *Structure* 21 1690–1697. 10.1016/j.str.2013.06.020 23932589 PMC3774310

[B16] Innis C. A. Shi J. Blundell T. L. (2000). Evolutionary trace analysis of TGF-beta and related growth factors: implications for site-directed mutagenesis. *Protein Eng.* 13 839–847. 10.1093/protein/13.12.839 11239083

[B17] Isogai E. Isogai H. Okumura K. Hori H. Tsuruta H. Kurebayashi Y. (2011). Tertiary structure-related activity of tick defensin (persulcatusin) in the taiga tick, Ixodes persulcatus. *Exp. Appl. Acarol.* 53 71–77. 10.1007/s10493-010-9379-3 20596886

[B18] Isogai E. H. Isogai H. Takahashi K. Kobayashi-Sakamoto M. Okumura K. (2009). Antimicrobial activity of three tick defensins and four mammalian cathelicidin-derived synthetic peptides against Lyme disease spirochetes and bacteria isolated from the midgut. *Exp. Appl. Acarol.* 49 221–228. 10.1007/s10493-009-9251-5 19229642

[B19] Izumiyama S. Omura M. Takasaki T. Ohmae H. Asahi H. (2009). Plasmodium falciparum: development and validation of a measure of intraerythrocytic growth using SYBR Green I in a flow cytometer. *Exp. Parasitol.* 121 144–150. 10.1016/j.exppara.2008.10.008 19017530

[B20] Jermann T. M. Opitz J. G. Stackhouse J. Benner S. A. (1995). Reconstructing the evolutionary history of the artiodactyl ribonuclease superfamily. *Nature* 374 57–59. 10.1038/374057a0 7532788

[B21] Jeyaprakash A. Hoy M. A. (2009). First divergence time estimate of spiders, scorpions, mites and ticks (subphylum: Chelicerata) inferred from mitochondrial phylogeny. *Exp. Appl. Acarol.* 47 1–18. 10.1007/s10493-008-9203-5 18931924

[B22] Katoh K. Standley D. M. (2013). MAFFT multiple sequence alignment software version 7: improvements in performance and usability. *Mol. Biol. Evol.* 30 772–780. 10.1093/molbev/mst010 23329690 PMC3603318

[B23] Kelly J. X. Smilkstein M. J. Brun R. Wittlin S. Cooper R. A. Lane K. D. (2009). Discovery of dual function acridones as a new antimalarial chemotype. *Nature* 459 270–273. 10.1038/nature07937 19357645 PMC8158239

[B24] Krysan D. J. Rockwell N. C. Fuller R. S. (1999). Quantitative characterization of furin specificity. Energetics of substrate discrimination using an internally consistent set of hexapeptidyl methylcoumarinamides. *J. Biol. Chem.* 274 23229–23234. 10.1074/jbc.274.33.23229 10438496

[B25] Le S. Gascuel O. (2008). An improved general amino acid replacement matrix. *Mol. Biol. Evol.* 25 1307–1320. 10.1093/molbev/msn067 18367465

[B26] Lomize M. A. Pogozheva I. D. Joo H. Mosberg H. I. Lomize A. L. (2012). OPM database and PPM web server: resources for positioning of proteins in membranes. *Nucleic Acids Res.* 40 D370–D376. 10.1093/nar/gkr703 21890895 PMC3245162

[B27] Malcolm B. A. Wilson K. P. Matthews B. W. Kirsch J. F. Wilson A. C. (1990). Ancestral lysozymes reconstructed, neutrality tested, and thermostability linked to hydrocarbon packing. *Nature* 345 86–89. 10.1038/345086a0 2330057

[B28] Marchler-Bauer A. Derbyshire M. K. Gonzales N. R. Lu S. Chitsaz F. Geer L. Y. (2015). CDD: NCBI’s conserved domain database. *Nuclei. Acids Res.* 43 D222–D226. 10.1093/nar/gku1221 PMC4383992 25414356

[B29] Mulder K. C. Lima L. A. Miranda V. Dias S. C. Franco O. L. (2013). Current scenario of peptide-based drugs: the key roles of cationic antitumor and antiviral peptides. *Front. Microbiol.* 4:321 10.3389/fmicb.2013.00321 PMC3813893 24198814

[B30] Mygind P. H. Fischer R. L. Schnorr K. M. Hansen M. T. Sönksen C. P. Ludvigsen S. (2005). Plectasin is a peptide antibiotic with therapeutic potential from a saprophytic fungus. *Nature* 437 975–980. 10.1038/nature04051 16222292

[B31] Nakajima Y. Ishibashi J. Yukuhiro F. Asaoka A. Taylor D. Yamakawa M. (2003). Antibacterial activity and mechanism of action of tick defensin against Gram-positive bacteria. *Biochim. Biophys. Acta* 1624 125–130. 10.1016/j.bbagen.2003.10.004 14642822

[B32] Nielsen R. (2002). Mapping mutations on phylogenies. *Syst. Biol.* 51 729–739. 10.1080/10635150290102393 12396587

[B33] Pauling L. Zuckerkandl E. (1963). Molecular “Restoration Studies” of extinct forms of life. *Acta Chem. Scand.* 17 S9–S16. 10.3891/acta.chem.scand.17s-0009

[B34] Petersen T. N. Brunak S. von Heijne G. Nielsen H. (2011). SignalP 4.0: discriminating signal peptides from transmembrane regions. *Nat. Methods* 8 785–786. 10.1038/nmeth.1701 21959131

[B35] Poon I. K. H. Baxter A. A. Lay F. T. Mills G. D. Adda C. G. Payne J. A. (2014). Phosphoinositide-mediated oligomerization of a defensin induces cell lysis. *Elife* 3:e01808 10.7554/eLife.01808 PMC3968744 24692446

[B36] Pupko T. Shamir I. Graur D. (2000). A fast algorithm for joint reconstruction of ancestral amino acid sequences. *Mol. Biol. Evol.* 17 890–896. 10.1093/oxfordjournals.molbev.a026369 10833195

[B37] Raman S. Vernon R. Thompson J. Tyka M. Sadreyev R. Pei J. (2009). Structure prediction for CASP8 with all-atom refinement using Rosetta. *Proteins* 77 89–99. 10.1002/prot.22540 19701941 PMC3688471

[B38] Schall J. J. Smith T. C. (2006). Detection of a malaria parasite (*Plasmodium mexicanum*) in ectoparasites (mites and ticks), and possible significance for transmission. *J. Parasitol.* 92 413–415. 10.1645/GE-688R.1 16729709

[B39] Schneider T. Kruse T. Wimmer R. Wiedemann I. Sass V. Pag U. (2010). Plectasin, a fungal defensin, targets the bacterial cell wall precursor lipid II. *Science* 328 1168–1172. 10.1126/science.1185723 20508130

[B40] Schrödinger L. (2010). *Maestro, version 9.1*. New York, NY: Schrödinger, LLC.

[B41] Tamura K. Stecher G. Peterson D. Filipski A. Kumar S. (2013). MEGA6: molecular evolutionary genetics analysis version 6.0. *Mol. Biol. Evol.* 30 2725–2729. 10.1093/molbev/mst197 24132122 PMC3840312

[B42] Thornton J. W. (2004). Resurrecting ancient genes: experimental analysis of extinct molecules. *Nat. Rev. Genet.* 5 366–375. 10.1038/nrg1324 15143319

[B43] Tian C. Gao B. Rodriguez M. C. Lanz-Mendoza H. Ma B. Zhu S. (2008). Gene expression, antiparasitic activity, and functional evolution of the drosomycin family. *Mol. Immunol.* 45 3909–3916. 10.1016/j.molimm.2008.06.025 18657321

[B44] Tonk M. Cabezas-Cruz A. Valdés J. J. Rego R. O. Chrudimská T. Strnad M. (2014a). Defensins from the tick *Ixodes scapularis* are effective against phytopathogenic fungi and the human bacterial pathogen *Listeria grayi*. *Parasit. Vectors* 7:554 10.1186/s13071-014-0554-y PMC4269947 25443032

[B45] Tonk M. Cabezas-Cruz A. Valdes J. J. Rego R. O. Rudenko N. Golovchenko M. (2014b). Identification and partial characterisation of new members of the *Ixodes ricinus* defensin family. *Gene.* 540 146–152. 10.1016/j.gene.2014.03.002 24607035

[B46] Tonk M. Cabezas-Cruz A. J. J. Valdés R. O. Rego L. Grubhoffer A. Estrada-Peña A. (2015). Ixodes ricinus defensins attack distantly-related pathogens. *Dev. Comp. Immunol* 53 358–365. 10.1016/j.dci.2015.08.001 26255244

[B47] Tsuji N. Battsetseg B. Boldbaatar D. Miyoshi T. Xuan X. Jr. Oliver J. H. (2007). Babesial vector tick defensin against *Babesia* sp. parasites. *Infect. Immun.* 75 3633–3640. 10.1128/IAI.00256-07 17485458 PMC1932947

[B48] Tufts D. M. Natarajan C. Revsbech I. G. Projecto-Garcia J. Hoffmann F. G. Weber R. E. (2015). Epistasis constrains mutational pathways of hemoglobin adaptation in high-altitude pikas. *Mol. Biol. Evol.* 32 287–298. 10.1093/molbev/msu311 25415962 PMC4298171

[B49] Uawonggul N. Thammasirirak S. Chaveerach A. Arkaravichien T. Bunyatratchata W. Ruangjirachuporn W. (2007). Purification and characterization of Heteroscorpine-1 (HS-1) toxin from *Heterometrus laoticus* scorpion venom. *Toxicon* 49 19–29. 10.1016/j.toxicon.2006.09.003 17056081

[B50] van Dijk A. Veldhuizen E. J. Kalkhove S. I. Tjeerdsma-van Bokhoven J. L. Romijn R. A. Haagsman H. P. (2007). The beta-defensin gallinacin-6 is expressed in the chicken digestive tract and has antimicrobial activity against food-borne pathogens. *Antimicrob. Agents Chemother.* 51 912–922. 10.1128/AAC.00568-06 17194828 PMC1803155

[B51] Veldhuizen E. J. Rijnders M. Claassen E. A. van Dijk A. Haagsman H. P. (2008). Porcine beta-defensin 2 displays broad antimicrobial activity against pathogenic intestinal bacteria. *Mol. Immunol.* 45 386–394. 10.1016/j.molimm.2007.06.001 17658606

[B52] Victor K. G. Cafiso D. S. (2001). Location and dynamics of basic peptides at the membrane interface: electron paramagnetic resonance spectroscopy of tetramethyl-piperidine-N-Oxyl-4-amino-4-carboxylic acid-labeled peptides. *Biophys. J.* 81 2241–2250. 10.1016/S0006-3495(01)75871-7 11566794 PMC1301695

[B53] Wang J. Bian G. Pan W. Feng T. Dai J. (2015). Molecular characterization of a defensin gene from a hard tick, *Dermacentor silvarum*. *Parasit. Vectors* 8:25 10.1186/s13071-014-0625-0 PMC4311433 25588982

[B54] Wang Y. Zhu S. (2011). The defensin gene family expansion in the tick *Ixodes scapularis*. *Dev. Comp. Immunol.* 35 1128–1134. 10.1016/j.dci.2011.03.030 21540051

[B55] Yang Z. Kumar S. Nei M. (1995). A new method of inference of ancestral nucleotide and amino acid sequences. *Genetics* 14 1641–1650. 10.1093/genetics/141.4.1641 PMC1206894 8601501

[B56] Yeaman M. R. Yount N. Y. (2007). Unifying themes in host defence effector polypeptides. *Nat. Rev. Microbiol.* 5 727–740. 10.1038/nrmicro1744 17703227

[B57] Yount N. Y. Yeaman M. R. (2004). Multidimensional signatures in antimicrobial peptides. *Proc. Natl. Acad. Sci. U.S.A.* 101 7363–7368. 10.1073/pnas.0401567101 15118082 PMC409924

[B58] Zhang Z. T. Zhu S. Y. (2009). Drosomycin, an essential component of antifungal defence in Drosophila. *Insect. Mol. Biol.* 18 549–556. 10.1111/j.1365-2583.2009.00907.x 19754735

[B59] Zhu S. Gao B. Tytgat J. (2005). Phylogenetic distribution, functional epitopes and evolution of the CSalphabeta superfamily. *Cell. Mol. Life Sci.* 62 2257–2269. 10.1007/s00018-005-5200-6 16143827 PMC11138386

[B60] Zhu S. Peigneur S. Gao B. Umetsu Y. Ohki S. Tytgat J. (2014). Experimental conversion of a defensin into a neurotoxin: implications for origin of toxic function. *Mol. Biol. Evol.* 31 546–559. 10.1093/molbev/msu038 24425781

