# Supplementary figures and images for: Antiplasmodial Activity Is an Ancient and Conserved Feature of Tick Defensins
Source: Front Microbiol. 2016 Oct 24;7:1682. doi: 10.3389/fmicb.2016.01682 (PMC5075766; doi:10.3389/fmicb.2016.01682)

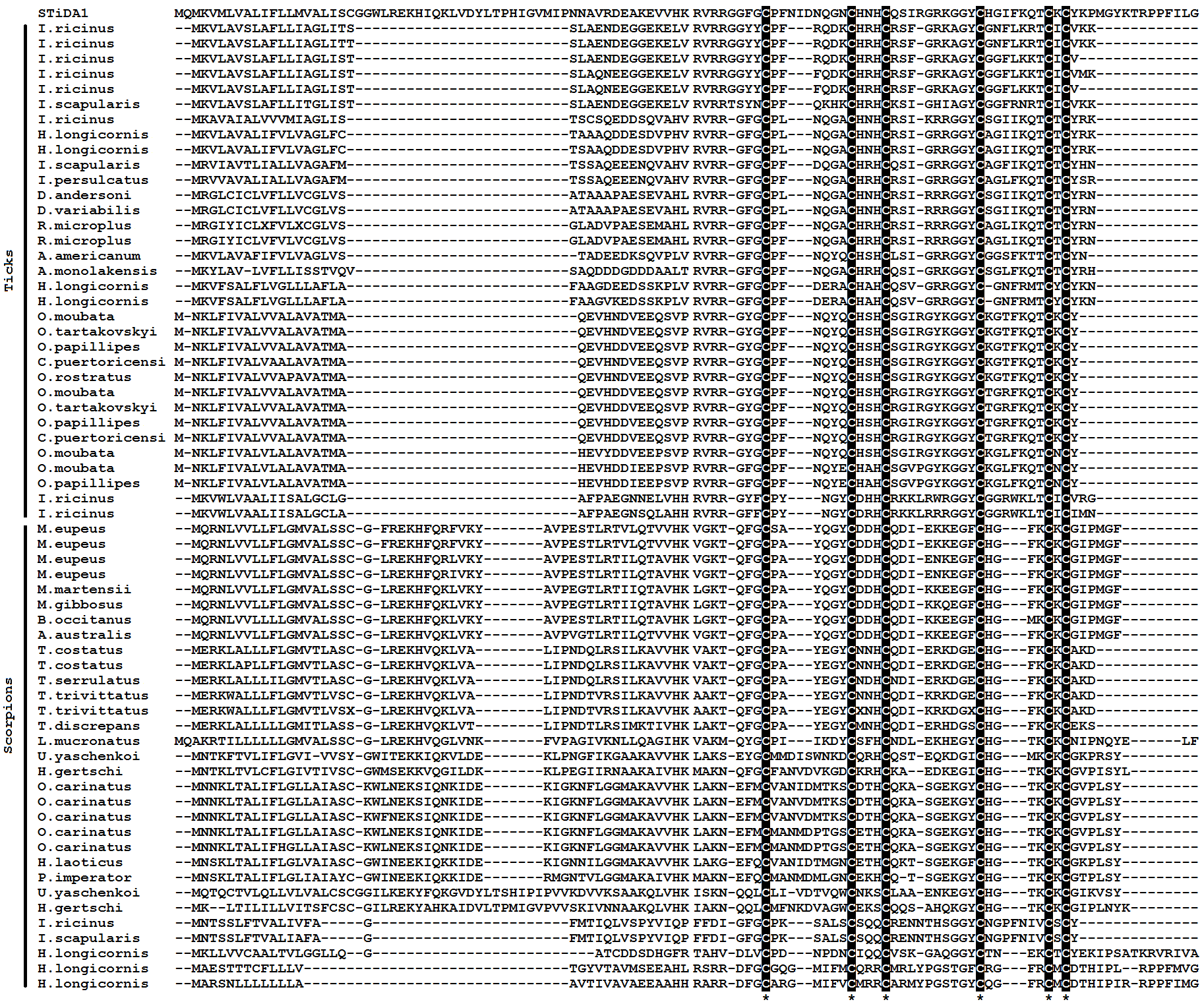

Supplement: Supplementary file 1 [file Image_1.TIFF]

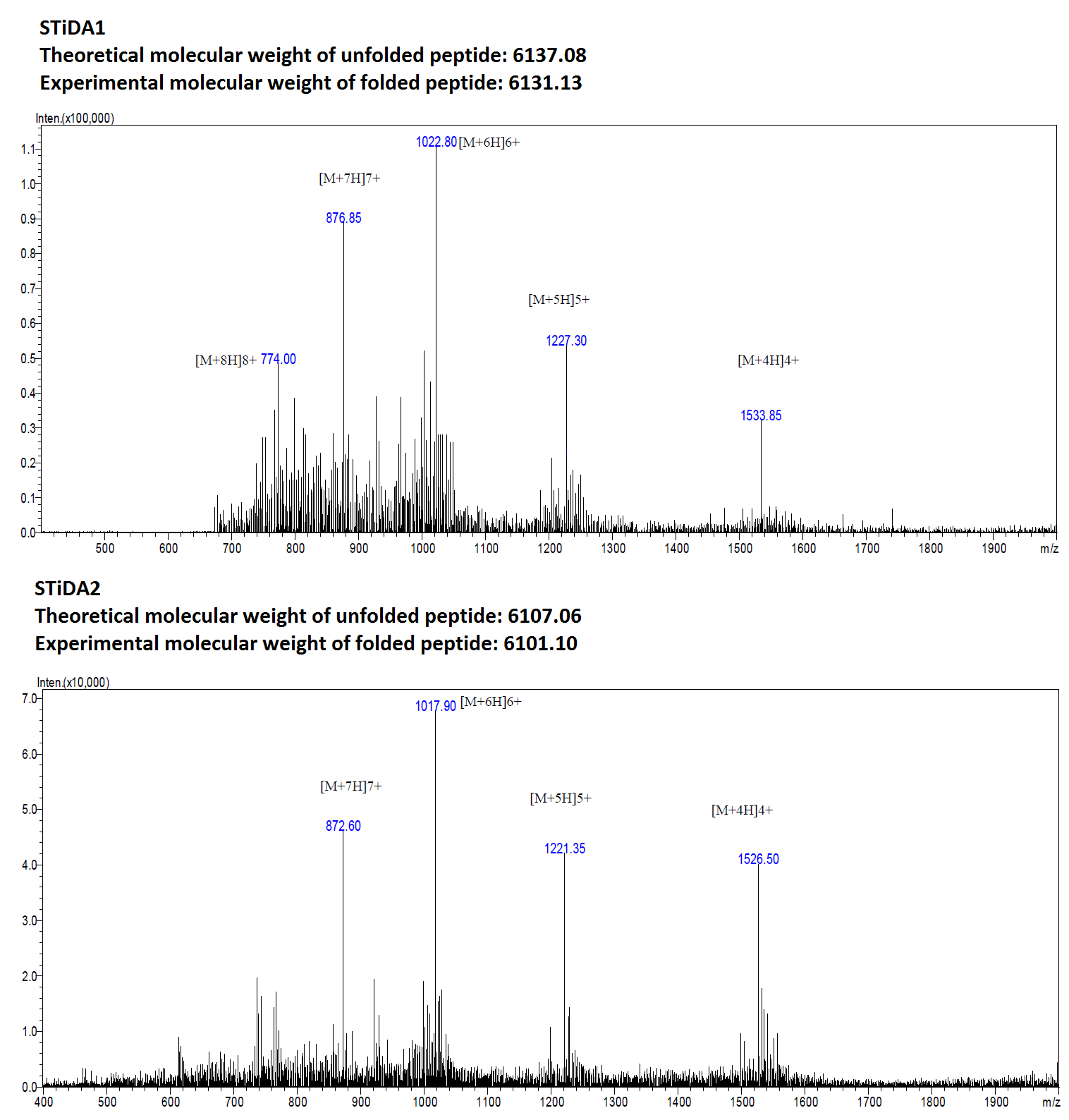

Supplement: Supplementary file 2 [file Image_2.TIFF]

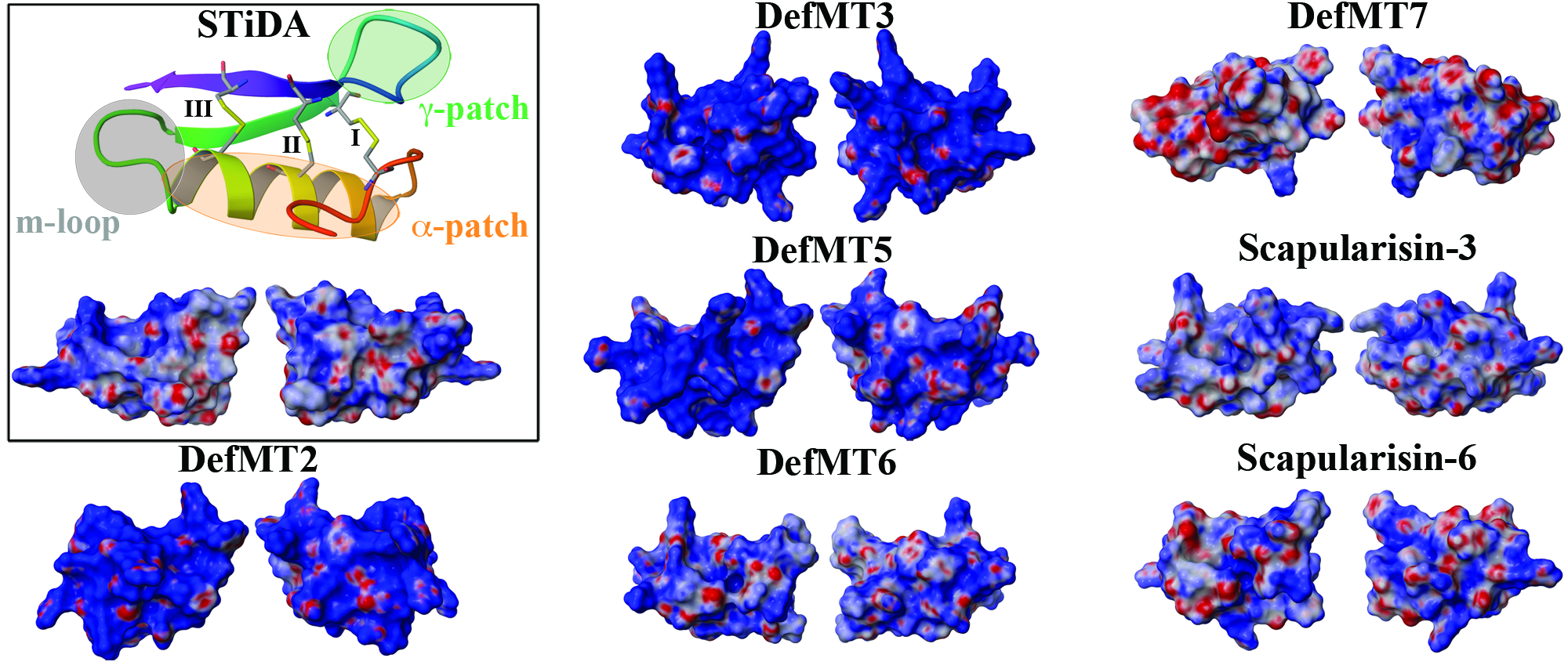

Supplement: Supplementary file 4 [file Image_4.TIF]
