# Supplementary material for: Antiplasmodial Activity Is an Ancient and Conserved Feature of Tick Defensins
Source: Front Microbiol. 2016 Oct 24;7:1682. doi: 10.3389/fmicb.2016.01682 (PMC5075766; doi:10.3389/fmicb.2016.01682)
Supplement: Supplementary file 5 [file Data_Sheet_1.PDF]

Supplementary file 1. Amino acid sequences of tick and scorpion defensins used for ancestral sequence reconstruction. Names of species and accession numbers for each sequence are shown.

Tick defensins

Ixodes ricinus.JAA653552(DefMT2)
MKVWLVAALIISALGCLGAFPAEGNNELVHHRVRRGYFCPYNGYCDHHCRKKLRWRGGYCGGRWKLTCICVRG

Ixodes ricinus.JAA71488(DefMT3)
MKVLAVSLAFLLIAGLITSSLAENDEGGEKELVRVRRGGYYCPFRQDKCHRHCRSFGRKAGYCGNFLKRTCICVKK

Ixodes ricinus.JAA71477(DefMT4)
MKVLAVSLAFLLIAGLITTSLAENDEGGEKELVRVRRGGYYCPFRQDKCHRHCRSFGRKAGYCGNFLKRTCICVKK

Ixodes ricinus.JAA66832(DefMT5)
MKVWLVAALIISALGCLAAFPAEGNSQLAHHRVRRGFFCPYNGYCDRHCRKKLRRRGGYCGGRWKLTCICIMN

Ixodes ricinus.JAA71516(DefMT6)
MKAVAIALVVVMIAGLISTSCSQEDDSQVAHVRVRRGFGCPLNQGACHNHCRSIKRRGGYCSGIIKQTCTCYRK

Ixodes ricinus.JAA69779(DefMT7)
MNTSSLFTVALIVFAGFMTIQLVSPYVIQPFFDIGFGCPKSALSCSQQCRENNTHSGGYCNGPFNIVCSCY

Ixodes ricinus.AY335442
MKVLAVSLAFLLIAGLISTSLAQNEEGGEKELVRVRRGGYYCPFFQDKCHRHCRSFGRKAGYCGGFLKKTCICVMK

Ixodes ricinus.EF067917
MKVLAVSLAFLLIAGLISTSLAQNEEGGEKELVRVRRGGYYCPFFQDKCHRHCRSFGRKAGYCGGFLKKTCICV

Ixodes ricinus.DQ361064
MKVLAVSLAFLLIAGLISTSLAENDEGGEKELVRVRRGGYYCPFRQDKCHRHCRSFGRKAGYCGGFLKKTCICV

Ixodes scapularis.AY660970
MRVIAVTLIALLVAGAFMTSSAQEEENQVAHVRVRRGFGCPFDQGACHRHCQSIGRRGGYCAGFIKQTCTCYHN

Ixodes scapularis.EEC17916
MNTSSLFTVALIAFAGFMTIQLVSPYVIQPFFDIGFGCPKSALSCSQQCRENNTHSGGYCNGPFNIVCSCY

Ixodes scapularis.EEC01374
MKVLAVSLAFLLITGLISTSLAENDEGGEKELVRVRRTSYNCPFQKHKCHRHCKSIGHIAGYCGGFRNRTCICVKK

Ixodes persulcatus.AB469201
MRVVAVALIALLVAGAFMTSSAQEEENQVAHVRVRRGFGCPFNQGACHRHCRSIGRRGGYCAGLFKQTCTCYSR

Haemaphysalis longicornis.AB105544
MKVLAVALIFVLVAGLFCTAAAQDDESDVPHVRVRRGFGCPLNQGACHNHCRSIGRRGGYCAGIIKQTCTCYRK

Haemaphysalis longicornis.EF432731
MKVFSALFLVGLLLAFLAFAAGDEEDSSKPLVRVRRGFGCPFDERACHAHCQSVGRRGGYCGNFRMTCYCYKN

Haemaphysalis longicornis.EU035972
MKVFSALFLVGLLLAFLAFAAGVKEDSSKPLVRVRRGFGCPFDERACHAHCQSVGRRGGYCGNFRMTCYCYKN

Haemaphysalis longicornis.EU035973
MKVLAVALIFVLVAGLFCTSAAQDDESDVPHVRVRRGFGCPLNQGACHNHCRSIGRRGGYCAGIIKQTCTCYRK

Haemaphysalis longicornis.ABO28926
MKLLVVCAALTVLGGLLQGATCDDSDHGFRTAHVDLVCPDNPDNCIQQCVSKGAQGGYCTNEKCTCYEKIPSATKRVRIVA

Haemaphysalis longicornis.EU627689
MAESTTTCFLLLVTGYVTAVMSEEAHLRSRRDFGCGQGMIFMCQRRCMRLYPGSTGFCRGFRCMCDTHIPLRPPFMVG

Haemaphysalis longicornis.HQ908088
MARSNLLLLLLLAAVTIVAVAEEAAHHRARRDFGCARGMIFVCMRRCARMYPGSTGYCQGFRCMCDTHIPIRRPPFIMG

Dermacentor andersoni.EF060192
MRGLCICLVFLLVCGLVSATAAAPAESEVAHLRVRRGFGCPLNQGACHNHCRSIRRRGGYCSGIIKQTCTCYRN

Dermacentor variabilis.AY181027
MRGLCICLVFLLVCGLVSATAAAPAESEVAHLRVRRGFGCPLNQGACHNHCRSIRRRGGYCSGIIKQTCTCYRN

Rhipicephalus microplus.AY233213
MRGIYICLXFVLXCGLVSGLADVPAESEMAHLRVRRGFGCPFNQGACHRHCRSIRRRGGYCAGLIKQTCTCYRN

Rhipicephalus microplus.Q86LE4
MRGIYICLVFVLVCGLVSGLADVPAESEMAHLRVRRGFGCPFNQGACHRHCRSIRRRGGYCAGLIKQTCTCYRN

Amblyomma americanum.DQ864986
MKVLAVAFIFVLVAGLVSTADEEDKSQVPLVRVRRGFGCPFNQYQCHSHCLSIGRRGGYCGGSFKTTCTCYN

Ornithodoros moubata.Q9BLJ3
MNKLFIVALVVALAVATMAQEVHNDVEEQSVPRVRRGYGCPFNQYQCHSHCSGIRGYKGGYCKGTFKQTCKCY

Ornithodoros moubata.Q9BLJ4
MNKLFIVALVVALAVATMAQEVHDDVEEQSVPRVRRGYGCPFNQYQCHSHCRGIRGYKGGYCTGRFKQTCKCY

Ornithodoros moubata.Q8MY08
MNKLFIVALVLALAVATMAHEVYDDVEEPSVPRVRRGYGCPFNQYQCHSHCSGIRGYKGGYCKGLFKQTCNCY

Ornithodoros moubata.Q8MY07
MNKLFIVALVLALAVATMAHEVHDDIEEPSVPRVRRGFGCPFNQYECHAHCSGVPGYKGGYCKGLFKQTCNCY

Ornithodoros tartakovskyi.ACJ04431
MNKLFIVALVVALAVATMAQEVHNDVEEQSVPRVRRGYGCPFNQYQCHSHCSGIRGYKGGYCKGTFKQTCKCY

Ornithodoros tartakovskyi.ACJ04432
MNKLFIVALVVALAVATMAQEVHDDVEEQSVPRVRRGYGCPFNQYQCHSHCRGIRGYKGGYCTGRFKQTCKCY

Ornithodoros papillipes.ACJ04425
MNKLFIVALVVALAVATMAQEVHDDVEEQSVPRVRRGYGCPFNQYQCHSHCSGIRGYKGGYCKGTFKQTCKCY

Ornithodoros papillipes.ACJ04426
MNKLFIVALVVALAVATMAQEVHDDVEEQSVPRVRRGYGCPFNQYQCHSHCRGIRGYKGGYCTGRFKQTCKCY

Ornithodoros papillipes.ACJ04427
MNKLFIVALVLALAVATMAHEVHDDIEEPSVPRVRRGFGCPFNQYECHAHCSGVPGYKGGYCKGLFKQTCNCY

Ornithodoros rostratus.ACJ04428
MNKLFIVALVVAPAVATMAQEVHNDVEEQSVPRVRRGYGCPFNQYQCHSHCSGIRGYKGGYCKGTFKQTCKCY

Carios puertoricensis.ACJ04429
MNKLFIVALVAALAVATMAQEVHNDVEEQSVPRVRRGYGCPFNQYQCHSHCSGIRGYKGGYCKGTFKQTCKCY

Carios puertoricensis.ACJ04430
MNKLFIVALVVALAVATMAQEVHDDVEEQSVPRVRRGYGCPFNQYQCHSHCRGIRGYKGGYCTGRFKQTCKCY

Argas monolakensis.Q09JJ7
MKYLAVLVFLLISSTVQVSAQDDDGDDDAALTRVRRGFGCPFNQGACHRHCQSIGRKGGYCSGLFKQTCTCYRH

Scorpion defensins

Mesobuthus eupeus.A9XE59
MQRNLVVLLFLGMVALSSCGFREKHFQRFVKYAVPESTLRTVLQTVVHKVGKTQFGCSAYQGYCDDHCQDIEKKEGFCHGFKC
KCGIPMGF

Mesobuthus eupeus.A9XE60
MQRNLVVLLFLGMVALSSCGFREKHFQRFVKYAVPESTLRTVLQTVVHKVGKTQFGCPAYQGYCDDHCQDIEKKEGFCHGFKC
KCGIPMGF

Mesobuthus eupeus.AIL48790
MQRNLVVLLFLGMVALSSCGLREKHFQRLVKYAVPESTLRTILQTAVHKVGKTQFGCPAYQGYCDDHCQDIENKEGFCHGFKC
KCGIPMGF

Mesobuthus eupeus.AIL48791
MQRNLVVLLFLGMVALSSCGLREKHFQRIVKYAVPESTLRTILQTAVHKVGKTQFGCPAYQGYCDDHCQDIENKEGFCHGFKCK
CGIPMGF

Mesobuthus martensii.Q9N661
MQRNLVVLLFLGMVALSSCGLREKHFQKLVKYAVPEGTLRTIIQTAVHKLGKTQFGCPAYQGYCDDHCQDIKKEEGFCHGFKCK
CGIPMGF

Mesobuthus gibbosus.AHZ63133
MQRNLVVLLFLGMVALSSCGLREKHFQKLVKYAVPEGTLRTIIQTAVHKLGKTQFGCPAYQGYCDDHCQDIKKQEGFCHGFKCK
CGIPMGF

Urodacus yaschenkoi.L0G8Z0
MNTKFTVLIFLGVIVVSYGWITEKKIQKVLDEKLPNGFIKGAAKAVVHKLAKSEYGCMMDISWNKDCQRHCQSTEQKDGICHG
MKCKCGKPRSY

Urodacus yaschenkoi.L0GCW2
MQTQCTVLQLLVLVALCSCGGILKEKYFQKGVDYLTSHIPIPVVKDVVKSAAKQLVHKISKNQQLCLIVDTVQWCNKSCLAAENK
EGYCHGTKCKCGIKVSY

Tityus costatus.Q5G8A6
MERKLALLLFLGMVTLASCGLREKHVQKLVALIPNDQLRSILKAVVHKVAKTQFGCPAYEGYCNNHCQDIERKDGECHGFKCKC
AKD

Tityus costatus.AAW72464
MERKLAPLLFLGMVTLASCGLREKHVQKLVALIPNDQLRSILKAVVHKVAKTQFGCPAYEGYCNNHCQDIERKDGECHGFKCKC
AKD

Tityus trivittatus.Q0GY46.
MERKWALLLFLGMVTLVSCGLREKHVQKLVALIPNDTVRSILKAVVHKAAKTQFGCPAYEGYCNNHCQDIKRKDGECHGFKCK
CAKD

Tityus trivittatus.ABE98261

MERKWALLLFLGMVTLVSXGLREKHVQKLVALIPNDTVRSILKAVVHKAAKTQFGCPAYEGYCXNHCQDIKRKDGXCXGFKCKC
AKD

Tityus serrulatus.P69940.
MERKLALLLILGMVTLASCGLREKHVQKLVALIPNDQLRSILKAVVHKVAKTQFGCPAYEGYCNDHCNDIERKDGECHGFKCKCA
KD

Tityus discrepans.Q0GY44
MERKLALLLLLGMITLASSGLREKHVQKLVTLIPNDTLRSIMKTIVHKLAKTQFGCPAYEGYCMNHCQDIERHDGSCHGFKCKCE
KS

Opistophthalmus carinatus.Q5WQZ9
MNNKLTALIFLGLLAIASCKWLNEKSIQNKIDEKIGKNFLGGMAKAVVHKLAKNEFMCVANIDMTKSCDTHCQKASGEKGYCH
GTKCKCGVPLSY

Opistophthalmus carinatus.Q5WR03
MNNKLTALIFLGLLAIASCKWFNEKSIQNKIDEKIGKNFLGGMAKAVVHKLAKNEFMCVANVDMTKSCDTHCQKASGEKGYCH
GTKCKCGVPLSY

Opistophthalmus carinatus.AAQ94353
MNNKLTALIFLGLLAIASCKWLNEKSIQNKIDEKIGKNFLGGMAKAVVHKLAKNEFMCMANMDPTGSCETHCQKASGEKGYC
HGTKCKCGVPLSY

Opistophthalmus carinatus.Q5WR01
MNNKLTALIFHGLLAIASCKWLNEKSIQNKIDEKIGKNFLGGMAKAVVHKLAKNEFMCMANMDPTGSCETHCQKASGEKGYC
HGTKCKCGVPLSY

Opistophthalmus carinatus.Q5WQZ7
MNNKLTALIFLGLLAIASCKWLNEKSIQNKIDEKIGKNFLGGMAKAVVHKLAKNEFMCVANVDMTKSCDTHCQKASGEKGYCH
GTKCKCGVPLSY

Hadrurus gertschi.Q0GY40
MNTKLTVLCFLGIVTIVSCGWMSEKKVQGILDKKLPEGIIRNAAKAIVHKMAKNQFGCFANVDVKGDCKRHCKAEDKEGICHGT
KCKCGVPISYL

Hadrurus gertschi.P0C8W5
MKLTILILLVITSFCSCGILREKYAHKAIDVLTPMIGVPVVSKIVNNAAKQLVHKIAKNQQLCMFNKDVAGWCEKSCQQSAHQKG
YCHGTKCKCGIPLNYK

Buthus occitanus israelis.B8XH40
MQRNLVVLLLLGMVALSSCGLREKHFQKLVKYAVPESTLRTILQTAVHKLGKTQFGCPAYQGYCDDHCQDIKKEEGFCHGMKC
KCGIPMGF

Lychas mucronatus.P0CI49
MQAKRTILLLLLLGMVALSSCGLREKHVQGLVNKFVPAGIVKNLLQAGIHKVAKMQYGCPIIKDYCSFHCNDLEKHEGYCHGTK
CKCNIPNQYELF

Androctonus australis.P69939
MQRNLVVLLFLGMVALSSCGLREKHVQKLVKYAVPVGTLRTILQTVVHKVGKTQFGCPAYQGYCDDHCQDIKKEEGFCHGFKC
KCGIPMGF

Heterometrus laoticus.P0C2F4
MNSKLTALIFLGLVAIASCGWINEEKIQKKIDEKIGNNILGGMAKAVVHKLAKGEFQCVANIDTMGNCETHCQKTSGEKGFCHG
TKCKCGKPLSY

Pandinus imperator.CAB96789

MNSKLTALIFLGLIAIAYCGWINEEKIQKKIDERMGNTVLGGMAKAIVHKMAKNEFQCMANMDMLGNCEKHCQTSGEKGYC
HGTKCKCGTPLSY
