# Supplementary material for: Antiplasmodial Activity Is an Ancient and Conserved Feature of Tick Defensins
Source: Front Microbiol. 2016 Oct 24;7:1682. doi: 10.3389/fmicb.2016.01682 (PMC5075766; doi:10.3389/fmicb.2016.01682)
Supplement: Supplementary file 6 [file Data_Sheet_2.PDF]

Supplementary file 2. Detailed materials and methods of Fmoc solid phase peptide synthesis (SPPS).

Peptide synthesis was performed on Wang resin as the solid support, using the base labile 9-fluorenylmethyloxycarbonyl (Fmoc) as protecting group.

Fmoc deprotection

0.08 mmol of Fmoc-Val-Wang resin were loaded into a fritted column equipped with a plastic cap. The resin was washed with 2 x 3-ml portions of dimethylformamide (DMF) for 1 minute each. About 3 ml of 20% piperidine were added in DMF and allowed the deprotection to continue for 15 minutes. During this time, gently swirl was applied to assure a complete mixing. After the reaction was completed (about 15 min.), the reaction column was drained and the resin was washed again with DMF (4 x 3ml).

Amide bond coupling

In a small vial, 3 equivalents Fmoc amino acid were pre-activated by combining it with 3 equivalents of HBTU, 6 equivalents of N,N'-Diisopropylethylamine (DIPEA), and 3 ml of DMF. The solution was fully dissolved and then allowed to react for an additional 3-5 minutes. Then, this coupling solution was added to the resin, and agitated every 2-3 minutes over a period of 20 minutes.

Cleavage

In order obtain the peptide in the free acid form, the ester linkage was cleaved using strongly acidic conditions such as Trifluoroacetic acid (TFA). Then, the resin was treated with 2-3 ml of a solution of TFA in water in a relation 95:5. The resin was gently agitated over a period of 25mins. Finally, the column was drained and the filtrate was carefully collected into a glass collection vessel.
