# Supplementary material for: Antiplasmodial Activity Is an Ancient and Conserved Feature of Tick Defensins
Source: Front Microbiol. 2016 Oct 24;7:1682. doi: 10.3389/fmicb.2016.01682 (PMC5075766; doi:10.3389/fmicb.2016.01682)
Supplement: Supplementary file 7 [file Data_Sheet_3.pdf]

Supplementary file 3. Site by site report of posterior probabilities.

Posterior probabilities (PB) for each site are shown.

Site	Reconstruction method					
	Joint	PB	Marginal	PB	Sample	PB
1	M	1.0	M	1.0	M	1.0
2	Q	0.24074	Q	0.24074	Q	0.29
3	M	1.0	M	1.0	M	1.0
4	K	1.0	K	1.0	K	1.0
5	V	0.998903	V	0.998903	V	1.0
6	M	0.997587	M	0.997587	M	0.99
7	L	0.999991	L	0.999991	L	1.0
8	V	1.0	V	1.0	V	1.0
9	A	0.999145	A	0.999145	A	1.0
10	L	1.0	L	1.0	L	1.0
11	I	0.996819	I	0.996819	I	1.0
12	F	1.0	F	1.0	F	1.0
13	L	1.0	L	1.0	L	1.0
14	L	1.0	L	1.0	L	1.0
15	M	1.0	M	1.0	M	1.0
16	V	1.0	V	1.0	V	1.0
17	A	0.986425	A	0.986425	A	0.98
18	L	1.0	L	1.0	L	1.0
19	I	0.815296	I	0.815296	I	0.74
20	S	1.0	S	1.0	S	1.0
21	C	1.0	C	1.0	C	1.0
22	G	0.444045	G	0.444045	G	0.43
23	G	1.0	G	1.0	G	1.0
24	W	1.0	W	1.0	W	1.0
25	L	1.0	L	1.0	L	1.0

26	R	1.0	R	1.0	R	1.0
27	E	1.0	E	1.0	E	1.0
28	K	1.0	K	1.0	K	1.0
29	H	1.0	H	1.0	H	1.0
30	I	0.999566	I	0.999566	I	1.0
31	Q	1.0	Q	1.0	Q	1.0
32	K	0.999999	K	0.999999	K	1.0
33	L	0.997007	L	0.997007	L	1.0
34	V	0.998369	V	0.998369	V	1.0
35	D	0.954272	D	0.954272	D	0.96
36	Y	0.999998	Y	0.999998	Y	1.0
37	L	0.87552	L	0.87552	L	0.85
38	T	0.521631	T	0.521631	T	0.49
39	P	0.394003	P	0.394003	P	0.36
40	H	0.155617	H	0.155617	H	0.24
41	I	0.551558	I	0.551558	I	0.62
42	G	0.294083	G	0.294083	G	0.34
43	V	0.473915	V	0.473915	V	0.48
44	M	0.500184	M	0.500184	A	0.56
45	I	0.98803	I	0.98803	I	0.99
46	P	1.0	P	1.0	P	1.0
47	N	0.997173	N	0.997173	N	1.0
48	N	0.936692	N	0.936692	N	0.96
49	A	0.994158	A	0.994158	A	0.98
50	V	0.994198	V	0.994198	V	0.97
51	R	0.92309	R	0.92309	R	0.96
52	D	0.998186	D	0.998186	D	1.0
53	E	0.987884	E	0.987884	E	0.99

54	A	0.685865	A	0.685865	A	0.7
55	K	0.519266	K	0.519266	K	0.53
56	E	1.0	E	1.0	E	1.0
57	V	1.0	V	1.0	V	1.0
58	V	1.0	V	1.0	V	1.0
59	H	1.0	H	1.0	H	1.0
60	K	1.0	K	1.0	K	1.0
61	R	1.0	R	1.0	R	1.0
62	V	1.0	V	1.0	V	1.0
63	R	1.0	R	1.0	R	1.0
64	R	1.0	R	1.0	R	1.0
65	G	0.999141	G	0.999141	G	1.0
66	G	0.999999	G	0.999999	G	1.0
67	F	1.0	F	1.0	F	1.0
68	G	1.0	G	1.0	G	1.0
69	C	1.0	C	1.0	C	1.0
70	P	1.0	P	1.0	P	1.0
71	F	1.0	F	1.0	F	1.0
72	N	0.978724	N	0.978724	N	0.99
73	I	0.52786	I	0.52786	I	0.46
74	D	0.993721	D	0.993721	D	1.0
75	N	0.999996	N	0.999996	N	1.0
76	Q	1.0	Q	1.0	Q	1.0
77	G	1.0	G	1.0	G	1.0
78	N	0.774025	N	0.774025	N	0.76
79	C	1.0	C	1.0	C	1.0
80	H	1.0	H	1.0	H	1.0
81	N	0.99996	N	0.99996	N	1.0

82	H	1.0	H	1.0	H	1.0
83	C	1.0	C	1.0	C	1.0
84	Q	0.999981	Q	0.999981	Q	1.0
85	S	0.97561	S	0.97561	S	0.98
86	I	1.0	I	1.0	I	1.0
87	R	0.999953	R	0.999953	R	1.0
88	G	1.0	G	1.0	G	1.0
89	R	1.0	R	1.0	R	1.0
90	K	0.999911	K	0.999911	K	1.0
91	G	0.999999	G	0.999999	G	1.0
92	G	1.0	G	1.0	G	1.0
93	Y	1.0	Y	1.0	Y	1.0
94	C	1.0	C	1.0	C	1.0
95	H	1.0	H	1.0	H	1.0
96	G	1.0	G	1.0	G	1.0
97	I	0.998804	I	0.998804	I	0.99
98	F	1.0	F	1.0	F	1.0
99	K	1.0	K	1.0	K	1.0
100	Q	0.930246	Q	0.930246	Q	0.92
101	T	0.988545	T	0.988545	T	0.95
102	C	1.0	C	1.0	C	1.0
103	K	1.0	K	1.0	K	1.0
104	C	1.0	C	1.0	C	1.0
105	Y	1.0	Y	1.0	Y	1.0
106	K	0.588151	K	0.588151	K	0.5
107	P	0.999989	P	0.999989	P	1.0
108	M	0.99996	M	0.99996	M	1.0
109	G	0.962733	G	0.962733	G	0.95

110	Y	0.978045	Y	0.978045	Y	0.98
111	K	0.369496	K	0.369496	K	0.42
112	T	0.125383	T	0.125383	A	0.16
113	R	0.575196	R	0.575196	R	0.64
114	P	0.610556	P	0.610556	P	0.58
115	P	0.573964	P	0.573964	P	0.59
116	F	0.693274	F	0.693274	F	0.68
117	I	0.306895	I	0.306895	I	0.34
118	L	0.535732	L	0.535732	L	0.61
119	G	0.553866	G	0.553866	G	0.53
